# Hemoglobin glycation index and short-term mortality in sepsis: a retrospective cohort study with external validation

**DOI:** 10.3389/fmed.2026.1859896

**Published:** 2026-07-08

**Authors:** Qianping Zhang, Yan Zhang, Xuemeng Li, Xinyi Tian, Zhijun Meng, Jin Zhang, Jie Weng, Kaifan Lin, Bihuan Cheng, Yuqiang Gong, Ye Gao

**Affiliations:** 1 Department of Anesthesia and Critical Care, The Second Affiliated Hospital of Wenzhou Medical University, Wenzhou, Zhejiang, China; 2 Ningbo Municipal Hospital of Traditional Chinese Medicine (TCM), Affiliated Hospital of Zhejiang Chinese Medical University, Ningbo, Zhejiang, China; 3 NHC Key Lab of Reproduction Regulation, Shanghai Engineering Research Center of Reproductive Health Drug and Devices, Shanghai Institute for Biomedical and Pharmaceutical Technologies, Shanghai, China; 4 The Second Clinical Medical College of Wenzhou Medical University, Wenzhou, Zhejiang, China; 5 Key Laboratory of Pediatric Anesthesiology, Ministry of Education, Wenzhou Medical University, Wenzhou, Zhejiang, China; 6 Laboratory of Anesthesiology of Zhejiang Province, The Second Affiliated Hospital and Yuying Children's Hospital of Wenzhou Medical University, Wenzhou, Zhejiang, China; 7 Department of General Practice, The Second Affiliated Hospital of Wenzhou Medical University, Wenzhou, China; 8 Wenzhou Key Laboratory of Precision General Practice and Health Management, Wenzhou, Zhejiang, China; 9 Wenzhou Key Laboratory of Precision General Practice and Health Management, Wenzhou, China

**Keywords:** critical care, hemoglobin glycation index, mortality, prognosis, sepsis

## Abstract

**Background:**

The hemoglobin glycation index (HGI) reflects interindividual variability in hemoglobin glycation beyond average glycemic exposure. Although stress-related dysglycemia is common in sepsis, the prognostic relevance of HGI in critically ill patients with sepsis remains unclear.

**Methods:**

We conducted a retrospective cohort study using the MIMIC-IV database (*n* = 2,073) as the primary cohort. HGI was calculated as the difference between observed and predicted HbA1c based on admission glucose. The primary outcome was 28-day mortality. Findings were validated in an independent external cohort (*n* = 166).

**Results:**

In the primary cohort, the 28-, 60-, and 90-day mortality rates were 23.25, 27.69, and 30.00%, respectively. Non-survivors exhibited significantly lower HGI levels than survivors (*p* < 0.001). Compared with the lowest HGI quartile (Q1), patients in the highest quartile (Q4) had a significantly lower risk of 28-day mortality (hazard ratio (HR) 0.70, 95% confidence interval (CI) 0.52–0.94; *p* = 0.018) and 60-day mortality (HR 0.76, 95% CI 0.58–1.00; *p* = 0.050). A similar but non-significant trend was observed for 90-day mortality (HR 0.80, 95% CI 0.62–1.04; *p* = 0.101). In the external validation cohort, higher HGI was similarly associated with reduced 28-day mortality, supporting the robustness of the primary findings.

**Conclusion:**

Higher HGI was associated with lower short-term mortality in this retrospective cohort of critically ill patients with sepsis, with the strongest evidence observed for 28-day mortality. These findings suggest that HGI may provide prognostic information during the early phase of sepsis. Prospective studies are needed to validate these findings and determine their clinical utility.

## Background

Sepsis is a life-threatening syndrome characterized by a dysregulated host response to infection, leading to systemic inflammation, immune dysfunction, and multi-organ failure (1). Despite advances in early recognition and therapeutic interventions, sepsis remains a leading cause of mortality worldwide (2). The complexity and heterogeneity of sepsis make accurate risk stratification challenging, necessitating the identification of reliable biomarkers to guide clinical management and improve patient outcomes (3).

Glycemic dysregulation plays a critical role in sepsis pathophysiology and prognosis. Both hyperglycemia and glucose variability have been associated with increased mortality and organ dysfunction in septic patients (4, 5). However, glucose-related risk in critically ill patients is not fully captured by a single glucose measurement. Early ICU blood glucose, as a snapshot of acute glycemic status, is strongly influenced by stress responses, inflammation, insulin resistance, nutritional support, and ICU interventions, and therefore may not distinguish stress-induced dysglycemia from pre-existing glycemic status. In contrast, hemoglobin A1c (HbA1c), a marker of longer-term glycemic exposure, has been widely used in diabetes assessment and has also been proposed as a prognostic indicator in sepsis (6–8). Nevertheless, HbA1c may not adequately capture acute illness-related metabolic instability, and individual variability in hemoglobin glycation may extend beyond blood glucose levels (9).

Recent studies in critical illness have therefore emphasized the prognostic value of glycemic metrics that go beyond isolated glucose or HbA1c measurements, including mean blood glucose, glycemic variability, glycemic gap, and hypoglycemia (10–14). For example, higher early glycemic variability within the first 24 h of ICU admission has been associated with increased 30-day mortality in septic patients (10), and both mean blood glucose and glycemic variability have been reported to correlate with ICU mortality in sepsis (11). In addition, glycemic gap-based approaches suggest that acute glucose levels should be interpreted in the context of chronic glycemic background (12). These findings support the concept that biomarkers integrating acute glucose status with longer-term glycemic exposure may provide additional prognostic information in septic patients.

HGI is a composite glycemic metric designed to quantify the discrepancy between observed HbA1c and HbA1c predicted from glucose levels. In the present study, HGI was calculated using early ICU blood glucose to estimate predicted HbA1c. Unlike isolated glucose or HbA1c measurements, HGI may capture interindividual differences in glycation tendency, erythrocyte biology, and the mismatch between acute glycemic stress and chronic glycemic exposure (15). Prior studies have shown that HGI is associated with cardiovascular disease and acute ischemic stroke in both diabetic and non-diabetic populations (16, 17).

However, whether HGI is associated with short-term mortality in critically ill patients with sepsis remains unclear. Therefore, this study aimed to investigate the association between HGI and short-term mortality in patients with sepsis and to evaluate the robustness of this association in an independent external validation cohort.

## Methods

### Data source and study population

This retrospective observational cohort study utilized data from the Medical Information Mart for Intensive Care IV (MIMIC-IV, version 3.1), a publicly accessible, de-identified critical care database. MIMIC-IV contains detailed information on more than 94,458 intensive care unit (ICU) admissions at Beth Israel Deaconess Medical Center in Boston, Massachusetts, collected between 2008 and 2022, including demographics, vital signs, laboratory measurements, and diagnostic information coded using both ICD-9 and ICD-10 systems.

One author (Qianping Zhang) completed the required training and obtained authorized access to the database (certification number: 46680798). As all data in MIMIC-IV are fully de-identified, individual informed consent was waived, and no additional ethical approval was required for this secondary analysis.

The external validation cohort was approved by the Medical Ethics Committee of the Second Affiliated Hospital of Wenzhou Medical University (ethics approval number: 2021-K-18-01). This study was conducted in accordance with the Reporting of studies Conducted using Observational Routinely-collected health Data (RECORD) guidelines (18).

### Inclusion and exclusion criteria

**Figure 1:**
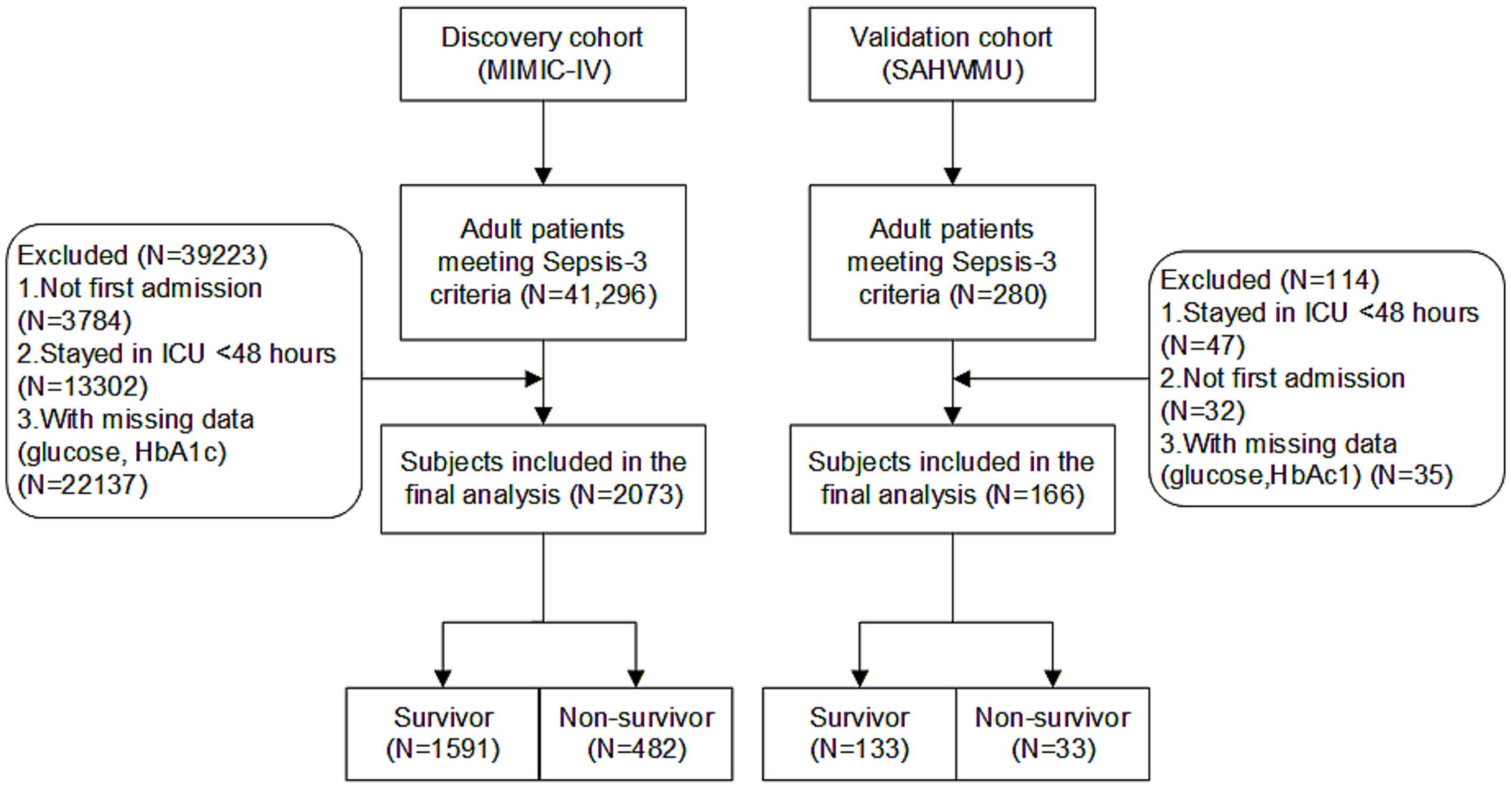
Flowchart of study participants.

Eligible participants were adult ICU patients with sepsis identified according to the Sepsis-3 criteria using the official MIMIC-IV derived sepsis3 table (mimiciv_derived. sepsis3), which is implemented in the publicly available MIT-LCP MIMIC code repository.^1^ Briefly, sepsis was defined as suspected infection accompanied by organ dysfunction, represented by a Sequential Organ Failure Assessment (SOFA) score ≥2 during ICU admission. ICD-9 and ICD-10 diagnosis codes were not used as the primary criterion for sepsis identification, but were used for comorbidity extraction and descriptive clinical characterization. Patients were excluded if they: (1) had multiple ICU admissions for sepsis, in which case only the first ICU admission was included; (2) had ICU stays shorter than 48 h; (3) lacked essential laboratory data, specifically HbA1c or early ICU blood glucose values; or (4) had more than 20% missing data points. Patient selection details are shown in Figure 1.

### Data collection and processing

In this study, data were extracted from the MIMIC-IV database using Structured Query Language (SQL) in PostgreSQL software (version 6.1) and Navicat Premium software (version 17.0.8). Demographic characteristics, comorbidities, laboratory test results, clinical interventions, and outcome data were obtained. Laboratory variables were defined as the first available measurements after ICU admission. Early ICU blood glucose was defined as the first available blood glucose measurement after ICU admission and was reported in mg/dL. HbA1c was defined as the first available HbA1c measurement after ICU admission and was reported as percentage (%). Follow-up started on the date of ICU admission and ended on the date of death or the predefined follow-up time point.

### Definition of exposure variables and outcome events

**Figure 2:**
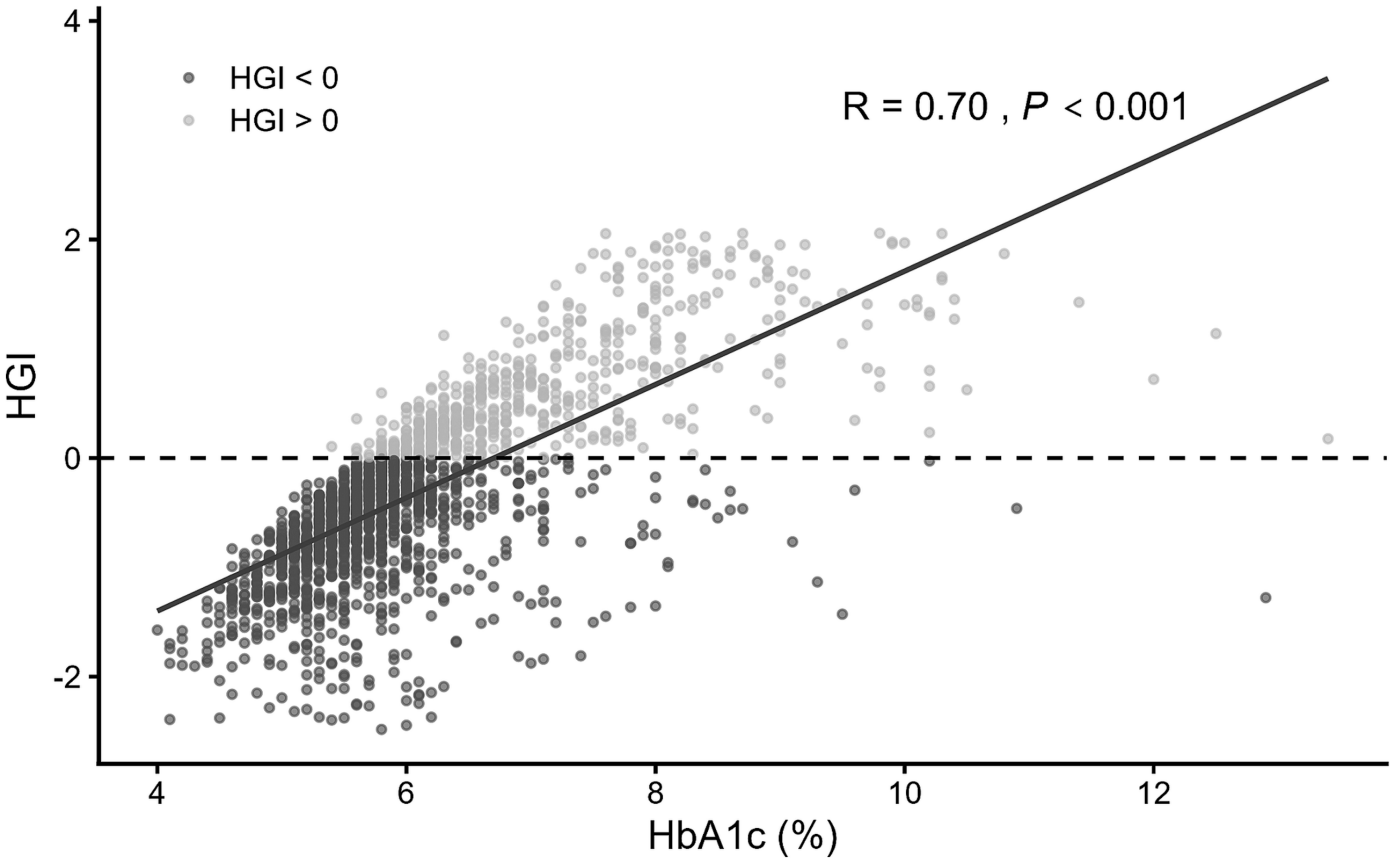
The correlation between HGI and HbA1c.

A linear regression model was fitted within the MIMIC-IV sepsis cohort, with observed HbA1c as the dependent variable and early ICU blood glucose as the independent variable. Based on this cohort-derived regression model, predicted HbA1c was calculated using the following equation: predicted HbA1c = 0.009 × early ICU blood glucose + 4.935. HGI was then calculated as observed HbA1c minus predicted HbA1c. To maintain a consistent exposure definition across cohorts, the same MIMIC-IV cohort-derived equation was applied to the external validation cohort. The correlation between observed HbA1c and HGI is illustrated in Figure 2.

The primary outcome of this study was all-cause mortality at 28 days post-ICU admission. Secondary outcomes were defined as all-cause mortality at 60 and 90 days following ICU admission.

### Statistical analysis

Categorical variables were analyzed using Fisher’s exact test or the chi-square test, as appropriate, and are reported as counts (percentages). Continuous variables were assessed using the Wilcoxon rank-sum test, Student’s *t*-test, or one-way analysis of variance (ANOVA).

To evaluate the association between the HGI and mortality risk at 28, 60, and 90 days, Cox proportional hazards regression models were constructed. Hazard ratios (HR) and corresponding 95% confidence intervals (CI) were calculated to quantify the impact of HGI on these outcomes. Three models were sequentially developed: Model 1 was unadjusted and included only HGI; Model 2 was adjusted for age, gender, race, and BMI; and Model 3 was additionally adjusted for covariates selected based on clinical expertise and existing literature (19). Specifically, Model 3 included age, gender, race, BMI, SOFA score, hypertension, diabetes, cerebrovascular disease, and mechanical ventilation. Variance inflation factors (VIFs) were computed to evaluate multicollinearity among the selected covariates. To further assess the robustness of the findings, two additional sensitivity models with extended covariate adjustment, Model 4 and Model 5, were constructed, and the corresponding results are presented in Supplementary Table S4. The proportional hazards assumption for the primary Cox models was assessed using Schoenfeld residuals. These diagnostics suggested no clear evidence of violation of the proportional hazards assumption. The corresponding Schoenfeld residual diagnostic plots are provided in Supplementary Figures S1, S2.

Patients with more than 20% missing data across the candidate variables were excluded before model construction. The primary multivariable Cox regression analyses were conducted using complete-case analysis according to the variables included in each specific model. Thus, patients with missing values in any exposure, outcome, or covariate required for a given model were excluded only from that specific model. The number and proportion of missing values for each variable included in the analysis are reported in Supplementary Table S3.

Kaplan–Meier survival curves were generated to visually depict differences in mortality according to HGI quartiles at each specified time point. Subgroup analyses were further conducted to evaluate the consistency of the associations between the HGI and mortality outcomes (28, 60, and 90 days) within predefined patient subpopulations. All statistical analyses were performed using R (version 4.1.2, R Foundation) and Python software. Statistical significance was defined as a two-tailed *p*-value of less than 0.05.

To further evaluate the robustness and generalizability of the primary findings, an independent external validation cohort was established. This cohort included 166 adult patients with sepsis admitted to the intensive care unit of the Second Affiliated Hospital of Wenzhou Medical University, all of whom met the Sepsis-3.0 diagnostic criteria.

In the external validation cohort, 28-day mortality were analyzed as outcomes due to follow-up availability. HGI was calculated using the same formula as in the primary cohort, and associations with mortality were evaluated using Cox proportional hazards models.

## Results

### Baseline characteristics of the study cohort

A total of 2,073 patients with sepsis were included in our study. The mortality rates at 28, 60, and 90 days were 23.25, 27.69, and 30.00%, respectively.

Table 1 presents comparisons between survivors and non-survivors during hospitalization. Non-survivors were older, had higher SOFA, and showed elevated inflammatory biomarkers, including WBC and blood urea nitrogen (BUN). Notably, HGI values were significantly lower among non-survivors compared to survivors (mean (SD): −0.21 (1.51) vs. 0.14 (1.67); *p* < 0.001), suggesting a potential prognostic association between HGI and clinical outcomes in patients with sepsis. Baseline characteristics and outcomes differed across HGI quartiles, as detailed in Supplementary Table S1.

**Table 1:** Baseline characteristics of the survivor and non-survivor groups.

Characteristics	Total (*n* = 2073)	Survivor (*n* = 1,591)	Non-survivor (*n* = 482)	*p*-value
Age (years), Median (IQR)	67.55 (56.79, 77.70)	66.35 (55.73, 76.54)	72.24 (61.15, 81.87)	<0.001
Male (%)	1,229 (59.29%)	954 (59.96%)	275 (57.05%)	0.278
BMI (kg/m^2^), Median (IQR)	28.15 (24.31, 33.53)	28.40 (24.52, 33.72)	27.23 (23.43, 32.38)	0.001
Race (%)				<0.001
White	1,085 (52.34%)	870 (54.68%)	215 (44.61%)	
Other/Unknown	988 (47.66%)	721 (45.32%)	267(55.39%)	
SOFA, Median (IQR)	3.00 (2.00, 4.00)	3.00 (2.00, 4.00)	3.00 (2.00, 4.00)	0.001
Charlson index, Median (IQR)	6.00 (4.00, 8.00)	5.00 (4.00, 7.00)	7.00 (4.00, 8.00)	<0.001
Hematocrit (%), Median (IQR)	32.90 (27.50, 37.80)	33.00 (27.65, 37.60)	32.65 (27.10, 38.10)	0.903
Hemoglobin (g/dL), Median (IQR)	10.90 (8.90, 12.40)	10.90 (9.00, 12.45)	10.70 (8.80, 12.40)	0.274
Platelets (K/uL), Median (IQR)	181.00 (135.00, 242.00)	182.00 (137.00, 245.00)	179.00 (126.25, 233.75)	0.050
WBC (K/uL), Median (IQR)	14.00 (10.30, 18.40)	13.80 (10.10, 18.10)	14.75 (10.93, 19.50)	<0.001
BUN (mg/dL), Median (IQR)	23.00 (16.00, 37.00)	22.00 (16.00, 35.00)	27.00 (18.00, 45.00)	<0.001
Creatinine (mg/dL), Median (IQR)	1.20 (0.90, 1.80)	1.20 (0.90, 1.70)	1.30 (1.00, 2.10)	<0.001
Sodium (mEq/L), Median (IQR)	137.00 (134.00, 140.00)	137.00 (134.00, 140.00)	137.50 (134.00, 141.00)	0.055
Potassium (mEq/L), Median (IQR)	3.80 (3.50, 4.20)	3.80 (3.50, 4.10)	3.80 (3.50, 4.20)	0.039
PT (sec), Median (IQR)	13.90 (12.50, 16.60)	13.80 (12.40, 16.20)	14.30 (12.72, 17.98)	<0.001
ALT (U/L), Median (IQR)	28.00 (17.00, 59.00)	28.00 (17.00, 56.00)	28.50 (16.00, 64.75)	0.414
ALP (U/L), Median (IQR)	82.00 (64.00, 110.00)	81.00 (63.00, 107.00)	88.00 (66.25, 120.75)	<0.001
AST (U/L), Median (IQR)	42.00 (25.00, 106.00)	41.00 (24.00, 94.50)	46.00 (26.00, 130.50)	0.010
HbA1c (%), Mean (SD)	6.57 (1.89)	6.63 (1.94)	6.38 (1.70)	0.010
Glucose (mg/dL), Mean (SD)	175.33 (103.19)	172.79 (98.43)	183.74 (117.24)	0.041
HGI, Mean (SD)	0.06 (1.64)	0.14 (1.67)	−0.21 (1.51)	<0.001
HR (bpm), Median (IQR)	104.00 (90.00, 119.00)	103.00 (91.00, 117.00)	105.00 (90.00, 122.00)	0.121
SBP (mmHg), Median (IQR)	90.00 (80.00, 102.00)	91.00 (80.00, 102.00)	89.00 (78.00, 101.75)	0.075
DBP (mmHg), Median (IQR)	47.00 (40.00, 54.00)	47.00 (40.00, 55.00)	46.00 (38.00, 52.00)	0.001
MBP (mmHg), Median (IQR)	61.00 (53.00, 68.00)	61.00 (54.00, 68.00)	60.00 (51.00, 66.88)	0.010
RR (breaths/min), Median (IQR)	28.00 (24.00, 32.00)	27.00 (24.00, 32.00)	28.00 (25.00, 33.00)	0.003
Temperature (°C), Median (IQR)	37.44 (37.06, 38.06)	37.44 (37.06, 38.00)	37.50 (37.00, 38.26)	0.133
SPO2 (%), Median (IQR)	93.00 (90.00, 95.00)	93.00 (90.00, 95.00)	93.00 (89.00, 95.00)	0.491
Ventilation (%)	1,186 (57.21%)	873 (54.87%)	313 (64.94%)	<0.001
CRRT (%)	167 (8.06%)	97 (6.10%)	70 (14.52%)	<0.001
Myocardial Infarct	602 (29.04%)	468 (29.42%)	134 (27.80%)	0.531
Congestive Heart failure (%)	788 (38.01%)	583 (36.64%)	205 (42.53%)	0.023
Cerebrovascular disease (%)	891 (42.98%)	635 (39.91%)	256 (53.11%)	<0.001
Chronic pulmonary disease (%)	437 (21.08%)	337 (21.18%)	100 (20.75%)	0.888
Renal disease	459 (22.14%)	340 (21.37%)	119 (24.69%)	0.140
Hypertension	1,080 (52.10%)	870 (54.68%)	210 (43.57%)	<0.001
Diabetes (%)	852 (41.10%)	675 (42.43%)	177 (36.72%)	0.029

Data are presented as mean (SD) for normally distributed variables (e.g., HGI, HbA1c) and median (IQR) for non-normally distributed variables.

### Relationship between the HGI and the endpoints

When HGI was analyzed by quartiles, higher HGI was associated with lower mortality. In the unadjusted model, compared with Q1, Q4 had lower risks of 28-day (HR 0.58, 95% CI 0.45–0.76; *p* < 0.001), 60-day (HR 0.60, 95% CI 0.47–0.76; *p* < 0.001), and 90-day mortality (HR 0.63, 95% CI 0.50–0.79; *p* < 0.001). These associations remained consistent after full adjustment (Model 3). The associations remained consistent after full adjustment for confounders in Model 3. Specifically, patients in the highest HGI quartile demonstrated significantly lower mortality risks at 28 days (HR: 0.70; 95% CI: 0.52–0.94; *p* = 0.018) and 60 days (HR: 0.76; 95% CI: 0.58–1.00; *p* = 0.050) compared to those in the lowest quartile. A similar protective trend was observed for 90-day mortality (HR: 0.80; 95% CI: 0.62–1.04; *p* = 0.101), although it did not reach statistical significance (Table 2). Restricted cubic spline analyses were also performed using HGI as a continuous variable, and the results are presented in Supplementary Figure S3.

**Table 2:** The association between HGI and all-cause mortality.

Exposure	Model 1		Model 2		Model 3	
	HR (95% CI)	*p*-value	HR (95% CI)	*p*-value	HR (95% CI)	*p*-value
28-day mortality						
HGI quartile						
Q1	1.0		1.0		1.0	
Q2	0.85 (0.67–1.07)	0.169	0.82 (0.64–1.04)	0.098	0.86 (0.68–1.09)	0.217
Q3	0.75 (0.59–0.96)	0.023	0.71 (0.56–0.91)	0.008	0.79 (0.61–1.01)	0.062
Q4	0.58 (0.45–0.76)	<0.001	0.61 (0.47–0.79)	<0.001	0.70 (0.52–0.94)	0.018
60-day mortality						
HGI quartile						
Q1	1.0		1.0		1.0	
Q2	0.85 (0.68–1.05)	0.133	0.82 (0.66–1.02)	0.074	0.85 (0.68–1.06)	0.158
Q3	0.77 (0.62–0.97)	0.025	0.73 (0.58–0.92)	0.007	0.81 (0.64–1.01)	0.065
Q4	0.60 (0.48–0.77)	<0.001	0.64 (0.51–0.82)	<0.001	0.76 (0.58–1.00)	0.050
90-day mortality						
HGI quartile						
Q1	1.0		1.0		1.0	
Q2	0.84 (0.68–1.03)	0.099	0.81 (0.65–1.00)	0.049	0.84 (0.68–1.05)	0.120
Q3	0.80 (0.65–1.00)	0.045	0.76 (0.61–0.94)	0.012	0.84 (0.67–1.04)	0.114
Q4	0.64 (0.51–0.80)	<0.001	0.67 (0.54–0.85)	<0.001	0.80 (0.62–1.04)	0.101

Model 1 was unadjusted, incorporating only the HGI; Model 2 was adjusted for age, gender, race, and BMI; Model 3 was adjusted for age, gender, race, BMI, SOFA score, hypertension; diabetes; cerebrovascular disease, ventilation.

**Figure 3:**
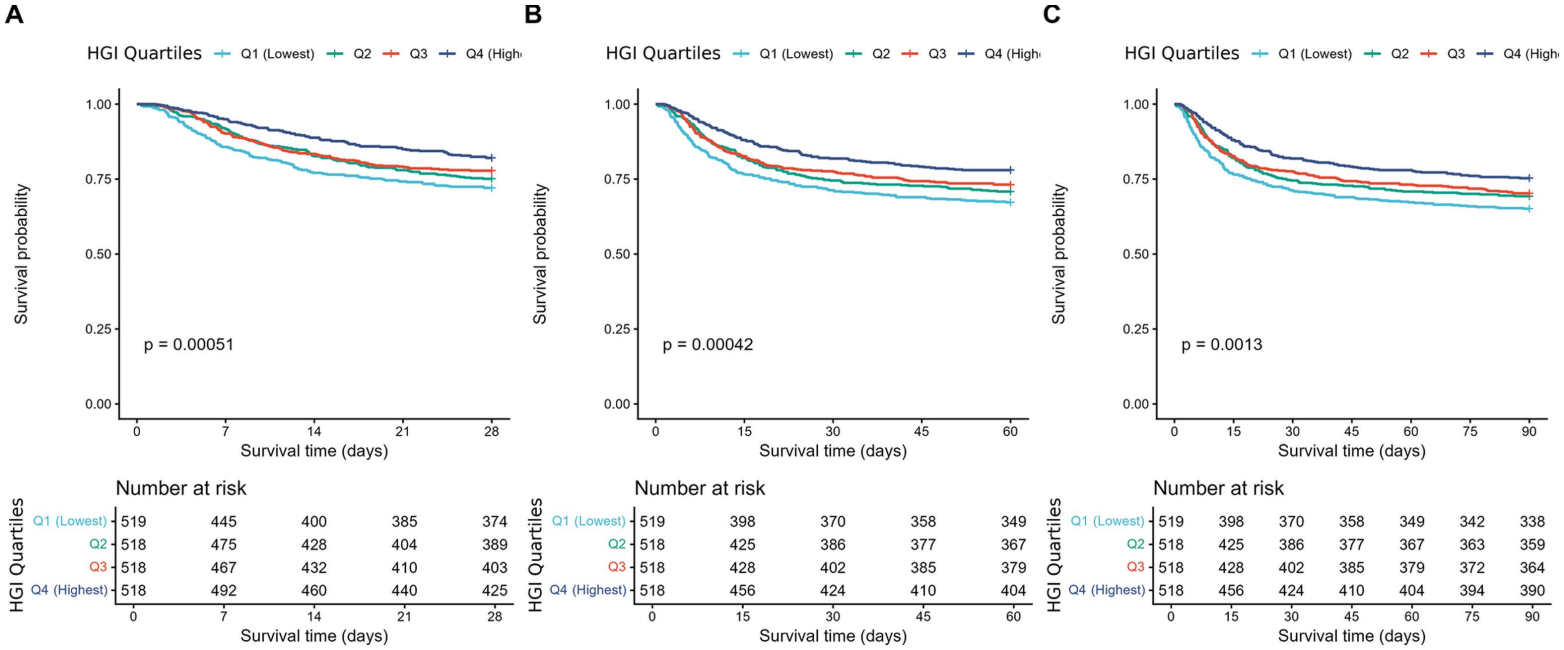
Comparison of 28-day **(A)**, 60-day **(B)**, and 90-day **(C)** mortality between HGI quartiles using Kaplan–Meier curves.

Additionally, Kaplan–Meier survival curves showed a stepwise increase in cumulative survival across HGI quartiles, with the highest HGI group (Q4) demonstrating significantly better survival at 28, 60, and 90 days (all log-rank *p* < 0.001). These findings suggest that a higher HGI was associated with improved survival in critically ill patients with sepsis (Figure 3).

### Subgroup analysis

**Figure 4:**
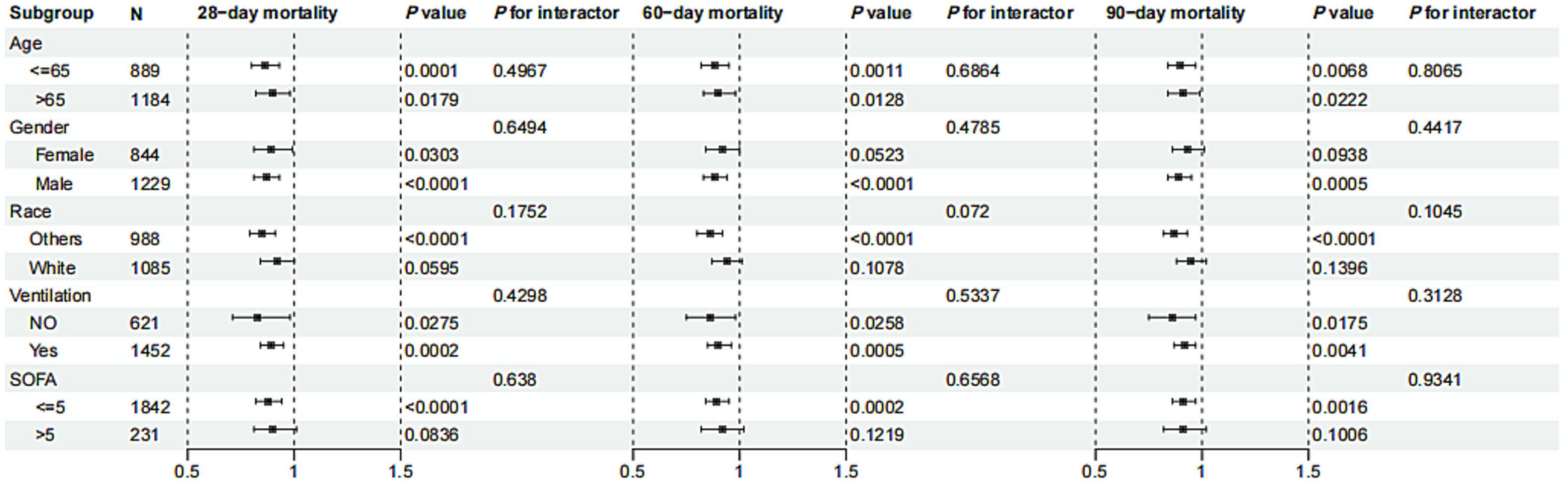
Forest plots of hazard ratios for the 28-day mortality in different subgroups.

In subgroup analyses, the association between HGI and mortality was further evaluated across clinically relevant strata, including age, gender, race, ventilation status, SOFA score, hypertension, diabetes, anemia, and CKD. No statistically significant interactions were observed across these subgroups, suggesting no clear evidence that these clinical characteristics substantially modified the association between HGI and short-term mortality. The main subgroup analyses are shown in Figure 4, and additional subgroup and interaction analyses for hypertension, diabetes, anemia, and CKD are presented in Supplementary Table S6.

### External validation of the association between HGI and mortality

In the external validation cohort, 166 patients were included and 33 deaths occurred within 28 days. When HGI was modeled as a continuous exposure in Cox proportional hazards models, the unadjusted analysis showed a non-significant trend toward a lower risk of 28-day mortality with higher HGI (Model 1: HR 0.80, 95% CI 0.61–1.06, *p* = 0.118). After adjustment for age and sex, this association remained borderline (Model 2: HR 0.78, 95% CI 0.58–1.05, *p* = 0.099). In the fully adjusted model further controlling for disease severity and comorbidities (age, sex, SOFA score), higher HGI was significantly associated with a reduced risk of 28-day mortality (Model 3: HR 0.59, 95% CI 0.43–0.81, *p* = 0.001). Notably, the direction and magnitude of the association were consistent with those observed in the primary cohort, supporting the robustness and external validity of the inverse relationship between HGI and short-term mortality (Table 3).

**Table 3:** The association between HGI and 28-day mortality in the external validation cohort.

Exposure	Model 1		Model 2		Model 3	
	HR (95% CI)	*p*-value	HR (95% CI)	*p*-value	HR (95% CI)	*p*-value
28-day mortality						
HGI	0.80 (0.61, 1.06)	0.118	0.78 (0.58, 1.05)	0.099	0.59 (0.43–0.81)	0.001

Model 1 was unadjusted, incorporating only the HGI; Model 2 was adjusted for age and gender; Model 3 was adjusted for age, gender, and SOFA score.

## Discussion

This study provides new evidence regarding the association between HGI and short-term mortality in critically ill patients with sepsis. Higher HGI was significantly associated with lower 28-day mortality after adjustment for potential confounders. The association was strongest for 28-day mortality, while the associations with 60- and 90-day mortality were weaker but directionally consistent. These findings suggest that HGI may provide early prognostic information in the acute phase of sepsis.

HGI has been widely investigated in diabetes, cardiovascular disease, and stroke populations (20, 21). In these chronic metabolic and vascular conditions, higher HGI has generally been associated with adverse outcomes. Previous studies from the DCCT, ACCORD, and AleCardio trials suggested that high HGI was related to increased risks of diabetes-related complications and cardiovascular mortality (15, 22, 23). Previous intensive glycemic control trials, including ACCORD and ADVANCE, also highlighted the complex relationship between glycemic control, glycation phenotype, and adverse cardiovascular outcomes in patients with type 2 diabetes (24, 25). In such settings, high HGI is often interpreted as a high-glycation phenotype, indicating that HbA1c is higher than expected for a given glucose level. This phenotype may reflect increased glycation susceptibility and greater accumulation of advanced glycation end products (AGEs), which can promote oxidative stress, inflammation, endothelial dysfunction, and vascular injury (26, 27).

Our findings appear to differ from this pattern, as higher HGI was associated with lower short-term mortality in patients with sepsis. This discrepancy may reflect the distinct biological context of critical illness. Unlike chronic metabolic diseases, sepsis is an acute systemic inflammatory state characterized by oxidative stress, endothelial injury, altered erythrocyte turnover, anemia, renal dysfunction, and frequent ICU interventions. Under these conditions, HGI may not solely represent chronic glycation propensity. Instead, lower HGI may partly reflect acute illness-related changes in HbA1c biology, such as shortened erythrocyte lifespan, erythrocyte injury, hemolysis, transfusion exposure, or impaired glycation dynamics, which could reduce observed HbA1c relative to early ICU blood glucose (27–31).

One possible explanation is that lower HGI in sepsis may reflect erythrocyte-related injury and altered glycation dynamics. Erythrocytes are vulnerable to oxidative damage during severe systemic inflammation. Inflammatory cytokines, including TNF-*α* and IL-6, can suppress erythropoiesis, while oxidative stress, excess nitric oxide, and complement activation may damage red blood cell membranes (32–35). These processes may promote erythrophagocytosis, premature erythrocyte clearance, and sepsis-associated anemia, thereby lowering HbA1c independently of actual glucose exposure and reducing HGI (28–30). Inflammatory conditions may also affect erythrocyte membrane structure, glucose transport, protein modification, deformability, and eryptosis, further influencing hemoglobin glycation (31). This interpretation should be considered a hypothesis rather than a confirmed biological mechanism.

In our cohort, patients in the lowest HGI quartile had lower hemoglobin levels than those in the highest quartile, which is compatible with this hypothesis. In addition, interindividual variability in glycation efficiency driven by genetic, enzymatic, and redox factors has been reported across racial and ethnic groups, supporting the concept that HGI reflects more than glycemic exposure alone (36). Nevertheless, this explanation remains hypothesis-generating rather than mechanistically proven, because direct measurements of erythrocyte lifespan, hemolysis, transfusion burden, erythrocyte deformability, and oxidative stress markers were unavailable.

Therefore, the inverse association between HGI and mortality observed in this study may represent a sepsis-specific prognostic signal rather than a direct contradiction of prior studies in chronic metabolic or cardiovascular populations. In chronic diseases, high HGI may primarily indicate increased glycation burden and vascular injury risk. In sepsis, however, low HGI may reflect acute disruption of erythrocyte and glycation biology, broader metabolic vulnerability, or critical illness-related hematologic stress. These contrasting patterns highlight the importance of interpreting HGI within disease-specific biological contexts.

Recent studies have also evaluated the prognostic relevance of HGI in sepsis, but the results remain inconsistent. He et al. reported that higher HGI was associated with increased 28-day and 365-day all-cause mortality in patients with sepsis from the MIMIC-IV database (37). In contrast, our study found that higher HGI was associated with lower short-term mortality, especially 28-day mortality. Xu et al. evaluated HGI combined with red cell distribution width and found that a higher HGI-RDW metric was associated with increased in-hospital mortality (38). These discrepancies may be related to differences in HGI calculation, exposure categorization, endpoint selection, adjustment strategy, study population, and whether HGI was analyzed alone or combined with erythrocyte-related markers. Therefore, the prognostic interpretation of HGI in sepsis may depend strongly on the analytic framework and clinical context.

The weaker associations observed for 60- and 90-day mortality may indicate that HGI primarily reflects early metabolic and hematologic vulnerability during the acute phase of sepsis. Longer-term outcomes are likely influenced by subsequent infection control, organ support, in-hospital complications, rehabilitation status, and post-discharge care, which may attenuate the prognostic contribution of baseline HGI over time.

Several limitations should be acknowledged. First, this was a retrospective observational study, and causality cannot be inferred. Residual confounding may persist despite multivariable adjustment. Second, although available hematologic and renal-related variables were considered, several non-glycemic factors may influence HbA1c and HGI, including anemia, hemolysis, red blood cell transfusion, altered erythrocyte lifespan, and chronic kidney disease. Direct measurements of these factors were not available, limiting mechanistic interpretation. Third, HGI depends on the equation used to estimate predicted HbA1c. Although the equation in this study was derived from the MIMIC-IV sepsis cohort and applied consistently to the external validation cohort, future studies should validate ICU-specific or sepsis-specific HGI formulas in independent prospective cohorts. Finally, because inclusion required both HbA1c and early ICU blood glucose measurements, selection bias cannot be excluded. Prospective studies with standardized glucose, HbA1c, hematologic, renal, and oxidative stress assessments are warranted to confirm our findings and clarify the biological mechanisms linking HGI to outcomes in sepsis.

## Conclusion

In conclusion, higher HGI was associated with lower short-term mortality in this retrospective cohort of critically ill patients with sepsis, with the strongest evidence observed for 28-day mortality. These findings warrant prospective confirmation in independent cohorts.

## Data Availability

The datasets used and analyzed during the current study are available from the MIMIC-IV database, a publicly accessible critical care database developed and maintained by the Laboratory for Computational Physiology at the Massachusetts Institute of Technology. Access to the database requires successful completion of the Collaborative Institutional Training Initiative (CITI) “Data or Specimens Only Research” course and adherence to the data use agreement. Further details on accessing the MIMIC-IV database can be found at https://physionet.org/content/mimiciv.

## Footnotes

1
https://github.com/MIT-LCP/mimic-code/blob/main/mimic-iv/concepts_postgres/sepsis/sepsis3.sql
