# Supplementary material for: Hemoglobin glycation index and short-term mortality in sepsis: a retrospective cohort study with external validation
Source: Front Med (Lausanne). 2026 Jul 8;13:1859896. doi: 10.3389/fmed.2026.1859896 (PMC13388289; doi:10.3389/fmed.2026.1859896)
Supplement: Supplementary file 1 [file Table_1.docx]

Table S1. Characteristics and outcomes of participants stratified by HGI status.

|  | Q1 (n=518) | Q2 (n=518) | Q3 (n=518) | Q4 (n=519) | *P* -value |
| --- | --- | --- | --- | --- | --- |
| Age (years) | 65.31 (53.97-75.53) | 69.16 (55.81-80.02) | 71.04 (61.54-80.49) | 65.27 (55.90-74.70) | <0.001 |
| Male (%) | 313 (60.42%) | 301 (58.11%) | 309 (59.65%) | 306 (58.96%) | 0.890 |
| BMI (kg/m2) | 26.85 (23.51-31.41) | 27.77 (23.94-32.84) | 28.41 (24.98-33.07) | 29.97 (25.35-35.98) | <0.001 |
| Race (%) |  |  |  |  | 0.457 |
| White | 268 (51.74%) | 280 (54.05%) | 279 (53.86%) | 258 (49.71%) |  |
| Other/Unknown | 250 (48.26%) | 238 (45.95%) | 239 (46.14%) | 261 (50.29%) |  |
| SOFA | 3.83 (2.13) | 3.37 (1.77) | 3.07 (1.61) | 3.23 (1.65) | <0.001 |
| Charlson Index | 5.00 (3.00-7.00) | 5.00 (3.00-7.00) | 6.00 (4.00-8.00) | 6.00 (4.00-8.00) | <0.001 |
| Hematocrit (%) | 30.40 (25.42-36.70) | 33.80 (28.72-38.10) | 33.65 (28.42-38.50) | 33.00 (28.10-37.55) | <0.001 |
| Hemoglobin (g/dL) | 10.10 (8.30-12.17) | 11.20 (9.40-12.50) | 11.00 (9.30-12.60) | 10.70 (9.00-12.40) | <0.001 |
| Platelets (K/uL) | 166.00 (115.00-229.50) | 181.00 (138.00-237.75) | 180.00 (138.25-237.00) | 196.00 (144.00-259.50) | <0.001 |
| WBC (K/uL) | 15.65 (11.60-20.28) | 13.90 (10.62-17.78) | 12.50 (9.30-17.00) | 14.00 (10.20-18.20) | <0.001 |
| BUN (mg/dL) | 24.00 (16.00-37.75) | 22.00 (16.00-34.75) | 22.00 (16.00-33.00) | 25.00 (16.00-45.00) | 0.015 |
| Creatinine (mg/dL) | 1.83 (2.06) | 1.66 (1.55) | 1.64 (1.62) | 1.78 (1.51) | <0.001 |
| Sodium (mEq/L) | 136.31 (6.33) | 137.25 (4.74) | 137.18 (5.25) | 136.34 (5.64) | 0.004 |
| Potassium (mEq/L) | 3.78 (0.57) | 3.80 (0.52) | 3.86 (0.54) | 3.87 (0.61) | 0.012 |
| PT (sec) | 14.45 (12.60-18.17) | 13.60 (12.40-15.80) | 14.10 (12.70-16.80) | 13.70 (12.30-16.00) | <0.001 |
| ALT (U/L) | 1.30 (1.20-1.70) | 1.20 (1.10-1.40) | 1.30 (1.20-1.50) | 1.20 (1.10-1.50) | <0.001 |
| ALP (U/L) | 30.00 (17.00-75.75) | 27.00 (16.00-62.75) | 28.00 (17.00-49.00) | 27.00 (17.00-53.00) | 0.069 |
| AST (U/L) | 82.00 (63.00-111.75) | 77.00 (63.00-102.00) | 81.00 (62.00-107.00) | 89.00 (69.00-122.00) | <0.001 |
| HbA1c (%) | 59.50 (29.00-159.00) | 42.00 (25.00-101.25) | 37.00 (24.00-78.00) | 38.00 (24.00-76.50) | <0.001 |
| Glucose (mg/dL) | 5.30 (5.00-5.70) | 5.60 (5.40-5.80) | 6.00 (5.80-6.38) | 8.40 (7.20-10.30) | <0.001 |
| HGI | 172.00 (131.25-242.75) | 130.00 (108.25-159.00) | 118.00 (104.00-150.00) | 184.00 (129.50-257.00) | <0.001 |
| HR (bpm) | 109.46 (22.21) | 104.77 (20.49) | 103.52 (20.61) | 105.54 (20.69) | <0.001 |
| SBP (mmHg) | 87.95 (19.47) | 92.59 (17.15) | 93.07 (17.39) | 91.76 (17.92) | <0.001 |
| DBP (mmHg) | 46.23 (11.20) | 47.87 (11.87) | 47.61 (11.21) | 46.89 (11.16) | 0.085 |
| MBP (mmHg) | 57.68 (15.83) | 60.22 (15.55) | 60.55 (14.55) | 59.35 (14.39) | 0.011 |
| RR (breaths/min) | 29.06 (6.76) | 28.49 (6.37) | 28.35 (6.24) | 28.76 (6.31) | 0.291 |
| Temperature (℃) | 37.50 (0.89) | 37.61 (0.84) | 37.59 (0.74) | 37.64 (0.84) | 0.038 |
| SPO2 (%) | 93.00 (90.00-95.00) | 93.00 (90.00-95.00) | 93.00 (90.00-95.00) | 93.00 (90.00-95.00) | 0.663 |
| ICU length of stay (days) | 6.21 (3.92-11.35) | 6.45 (3.89-10.85) | 5.86 (3.86-9.91) | 6.44 (3.75-12.32) | 0.170 |
| Hospital length of stay (days) | 12.59 (7.61-20.77) | 12.28 (7.80-20.25) | 11.85 (7.07-19.67) | 13.96 (8.39-22.96) | 0.053 |
| Hospital mortality | 121 (23.36%) | 98 (18.92%) | 106 (20.46%) | 83 (15.99%) | 0.026 |
| 28-day mortality | 146 (28.19%) | 127 (24.52%) | 116 (22.39%) | 93 (17.92%) | 0.001 |
| 60-day mortality | 171 (33.01%) | 149 (28.76%) | 140 (27.03%) | 114 (21.97%) | 0.001 |
| 90-day mortality | 182 (35.14%) | 158 (30.50%) | 154 (29.73%) | 128 (24.66%) | 0.003 |
| Ventilation (%) | 189 (57.10%) | 176 (53.33%) | 167 (50.30%) | 170 (51.20%) | 0.302 |
| Myocardial infarct (%) | 154 (29.73%) | 133 (25.68%) | 160 (30.89%) | 155 (29.87%) | 0.262 |
| Congestive heart failure (%) | 185 (35.71%) | 181 (34.94%) | 222 (42.86%) | 200 (38.54%) | 0.038 |
| Cerebrovascular disease (%) | 214 (41.31%) | 243 (46.91%) | 236 (45.56%) | 198 (38.15%) | 0.017 |
| Chronic pulmonary disease (%) | 101 (19.50%) | 103 (19.88%) | 126 (24.32%) | 107 (20.62%) | 0.206 |
| Renal disease (%) | 112 (21.62%) | 97 (18.73%) | 106 (20.46%) | 144 (27.75%) | 0.003 |
| Hypertension (%) | 239 (46.14%) | 267 (51.54%) | 289 (55.79%) | 285 (54.91%) | 0.008 |
| Diabetes (%) | 118 (22.78%) | 116 (22.39%) | 167 (32.24%) | 451 (86.90%) | <0.001 |

Continuous variables are expressed as mean (SD) or median (IQR), as appropriate. Categorical variables are expressed as count (%). For mortality outcomes, percentages represent within-quartile mortality rates, calculated using the number of patients in each HGI quartile as the denominator.

Table S2. Baseline characteristics of the included and excluded patients.

|  | Excluded (n= 22137) | Included (n= 2073) | *P* -value |
| --- | --- | --- | --- |
| Age (years) | 66.38 (16.13) | 66.34 (15.23) | 0.92 |
| Male (%) | 12605 (56.94) | 1229 (59.29) | 0.04 |
| Race (%) |  |  | <0.01 |
| White | 14595 (65.93) | 1085 (52.34) |  |
| Other/Unknown | 721 (45.32%) | 267(55.39%) |  |
| SOFA | 3.81 (2.09) | 3.37 (1.82) | <0.01 |
| Charlson index | 5.16 (2.91) | 5.70 (2.87) | <0.01 |
| Hematocrit (%) | 34.30 (6.46) | 32.74 (7.04) | <0.01 |
| Hemoglobin (g/dL) | 11.16 (2.19) | 10.75 (2.38) | <0.01 |
| Platelets (K/uL) | 227.93 (125.10) | 194.31 (93.22) | <0.01 |
| WBC (K/uL) | 15.88 (12.88) | 15.49 (9.71) | 0.18 |
| BUN (mg/dL) | 34.22 (26.57) | 30.95 (23.71) | <0.01 |
| Creatinine (mg/dL) | 1.89 (1.95) | 1.73 (1.75) | <0.01 |
| Sodium (mEq/L) | 139.93 (5.59) | 136.77 (5.53) | <0.01 |
| Potassium (mEq/L) | 4.71 (0.92) | 3.83 (0.56) | <0.01 |
| PT (sec) | 19.14 (13.53) | 16.47 (9.74) | <0.01 |
| ALT (U/L) | 189.21 (913.91) | 144.45 (784.10) | 0.03 |
| ALP (U/L) | 132.76 (147.09) | 101.49 (89.90) | <0.01 |
| AST (U/L) | 330.96 (1437.28) | 251.74 (1426.49) | 0.02 |
| HR (bpm) | 107.68 (21.83) | 105.82 (21.12) | <0.01 |
| SBP (mmHg) | 147.83 (23.93) | 91.34 (18.10) | <0.01 |
| DBP (mmHg) | 87.92 (20.86) | 47.15 (11.38) | <0.01 |
| MBP (mmHg) | 106.34 (28.11) | 59.45 (15.12) | <0.01 |
| RR (breaths/min) | 28.97 (6.86) | 28.66 (6.42) | 0.05 |
| Temperature (℃) | 37.55 (0.83) | 37.58 (0.83) | 0.07 |
| SPO2 (%) | 99.61 (0.99) | 91.77 (6.09) | <0.01 |
| Ventilation (%) | 12974 (58.61) | 1186 (57.21) | 0.23 |
| CRRT (%) | 3196 (14.44) | 167 (8.06) | <0.01 |
| Myocardial Infarct (%) | 3337 (15.07) | 602 (29.04) | <0.01 |
| Congestive Heart failure (%) | 6764 (30.56) | 788 (38.01) | <0.01 |
| Cerebrovascular disease (%) | 2937 (13.27) | 891 (42.98) | <0.01 |
| Chronic pulmonary disease (%) | 5990 (27.06) | 437 (21.08) | <0.01 |
| Renal disease (%) | 5546 (25.05) | 459 (22.14) | <0.01 |
| Hypertension (%) | 8262 (37.32) | 1080 (52.10) | <0.01 |
| Diabetes (%) | 7048 (31.84) | 852 (41.10) | <0.01 |

Continuous variables are expressed as mean (SD); Categorical variables are expressed as count (%)

Table S3. Summary of missing values for baseline covariates

| Variable | Missing Count | Missing Rate |
| --- | --- | --- |
| Male (%) | 0 | 0.00% |
| Race (%) | 0 | 0.00% |
| SOFA | 0 | 0.00% |
| Charlson index | 0 | 0.00% |
| Hematocrit (%) | 1 | 0.05% |
| Hemoglobin (g/dL) | 2 | 0.10% |
| Platelets (K/uL) | 2 | 0.10% |
| WBC (K/uL) | 1 | 0.05% |
| BUN (mg/dL) | 0 | 0.00% |
| Creatinine (mg/dL) | 0 | 0.00% |
| Sodium (mEq/L) | 1 | 0.05% |
| Potassium (mEq/L) | 1 | 0.05% |
| PT (sec) | 52 | 2.51% |
| ALT (U/L) | 352 | 16.98% |
| ALP (U/L) | 361 | 17.41% |
| AST (U/L) | 347 | 16.74% |
| HR (bpm) | 2 | 0.10% |
| SBP (mmHg) | 3 | 0.14% |
| DBP (mmHg) | 3 | 0.14% |
| MBP (mmHg) | 2 | 0.10% |
| RR (breaths/min) | 5 | 0.24% |
| Temperature (℃) | 59 | 2.85% |
| SPO2 (%) | 3 | 0.14% |
| Ventilation (%) | 124 | 5.98% |
| Myocardial Infarct (%) | 0 | 0.00% |
| Congestive Heart failure (%) | 0 | 0.00% |
| Cerebrovascular disease (%) | 0 | 0.00% |
| Chronic pulmonary disease (%) | 0 | 0.00% |
| Renal disease (%) | 0 | 0.00% |
| Hypertension (%) | 0 | 0.00% |
| Diabetes (%) | 0 | 0.00% |

Supplementary Table S4. Sensitivity analyses using extended and alternative severity-adjusted models

| **Exposure** | **Model 4** |  | **Model 5** |  |
| --- | --- | --- | --- | --- |
|  | HR (95% CI) | *P -*value | HR (95% CI) | *P -*value |
| **28-day Mortality** |  |  |  |  |
| HGI quartile |  |  |  |  |
| Q1 | 1.0 | — | 1.0 | — |
| Q2 | 0.91 (0.71–1.15) | 0.423 | 0.96 (0.76–1.23) | 0.767 |
| Q3 | 0.79 (0.62–1.02) | 0.069 | 0.87 (0.68–1.12) | 0.281 |
| Q4 | 0.61 (0.46–0.79) | <0.001 | 0.61 (0.46–0.79) | <0.001 |
| **60-day Mortality** |  |  |  |  |
| HGI quartile |  |  |  |  |
| Q1 | 1.0 | — | 1.0 | — |
| Q2 | 0.91 (0.73–1.13) | 0.391 | 0.96 (0.77–1.20) | 0.744 |
| Q3 | 0.81 (0.64–1.02) | 0.068 | 0.88 (0.70–1.11) | 0.291 |
| Q4 | 0.64 (0.50–0.81) | <0.001 | 0.63 (0.49–0.80) | <0.001 |
| **90-day Mortality** |  |  |  |  |
| HGI quartile |  |  |  |  |
| Q1 | 1.0 | — | 1.0 | — |
| Q2 | 0.90 (0.72–1.11) | 0.332 | 0.95 (0.77–1.18) | 0.657 |
| Q3 | 0.84 (0.67–1.04) | 0.109 | 0.91 (0.73–1.13) | 0.401 |
| Q4 | 0.67 (0.53–0.84) | <0.001 | 0.66 (0.52–0.83) | <0.001 |

Model 4 was the extended adjustment model, adjusted for age, gender, race, BMI, SOFA score, Charlson comorbidity index, mechanical ventilation, vasoactive drug use, and continuous renal replacement therapy.

Model 5 was the alternative severity-adjusted model, adjusted for age, gender, race, BMI, APSIII, Charlson comorbidity index, mechanical ventilation, vasoactive drug use, and continuous renal replacement therapy.

Q1 was used as the reference group.

HGI, hemoglobin glycation index; HR, hazard ratio; CI, confidence interval; BMI, body mass index; SOFA, Sequential Organ Failure Assessment; APSIII, Acute Physiology Score III; CRRT, continuous renal replacement therapy.

Supplementary Table S5. Distribution of SOFA score categories by 28-day mortality

| SOFA score category | Survivors, n (%) | Non-survivors, n (%) |
| --- | --- | --- |
| 0–2 | 721 (45.3%) | 189 (39.2%) |
| 3–4 | 598 (37.6%) | 180 (37.3%) |
| 5–6 | 176 (11.1%) | 70 (14.5%) |
| ≥7 | 96 (6.0%) | 43 (8.9%) |

Supplementary Table S6. Sensitivity analyses stratified by diabetes, anemia, CKD and hypertension.

| Subgroup | N | HR (95% CI) | *P* -value | *P* for interactor |
| --- | --- | --- | --- | --- |
| diabetes |  | |  | 0.654 |
| No | 1221 | 0.93 (0.83–1.04) | 0.182 |  |
| Yes | 852 | 0.91 (0.83–0.99) | 0.030 |  |
| anemia |  | |  | 0.367 |
| No | 484 | 0.89 (0.75–1.06) | 0.190 |  |
| Yes | 1589 | 0.91 (0.84–0.98) | 0.009 |  |
| CKD |  | |  | 0.793 |
| No | 1614 | 0.92 (0.85–1.00) | 0.064 |  |
| Yes | 459 | 0.87 (0.78–0.97) | 0.013 |  |
| hypertension |  | |  | 0.161 |
| No | 993 | 0.88 (0.82–0.94) | 0.001 |  |
| Yes | 1080 | 1.01 (0.90–1.13) | 0.864 |  |

Supplementary Table S7: Baseline characteristics of the external validation cohort stratified by 28-day mortality status.

| Variable | Overall | Survivors | Non-survivors | *P* -value |
| --- | --- | --- | --- | --- |
| Age, years | 70 (57, 79) | 70 (57, 79) | 71 (59, 81) | 0.50 |
| Sex |  |  |  | 0.90 |
| Female | 60 (37.74%) | 48 (36.09%) | 12 (36.36%) |  |
| Male | 106 (62.26%) | 85 (63.91%) | 21 (63.64%) |  |
| HbA1c | 5.95 (5.37, 7.29) | 6.00 (5.38, 7.60) | 5.72 (5.36, 6.36) | 0.20 |
| Glucose | 7.64 (6.43, 10.34) | 7.68 (6.45, 10.39) | 6.91 (6.30, 8.95) | 0.20 |
| SOFA score | 7.00 (5.00, 10.00) | 6.00 (4.00, 9.00) | 10.00 (6.00, 12.00) | 0.003 |
| APACHE II score | 18.00 (12.00, 22.00) | 17.00 (11.00, 21.00) | 23.00 (19.00, 28.00) | <0.001 |
| Charlson comorbidity index | 6.00 (4.00, 8.00) | 6.00 (4.00, 7.00) | 6.00 (4.00, 8.00) | 0.40 |
| Lactate | 1.40 (1.10, 2.20) | 1.30 (1.10, 1.90) | 1.90 (1.25, 5.90) | <0.001 |
| Hemoglobin | 102.50 (84.00, 120.00) | 103.00 (86.00, 122.00) | 101.00 (80.00, 113.00) | 0.20 |
| Creatinine | 87.45 (64.90, 150.80) | 86.00 (64.60, 135.60) | 104.00 (67.90, 222.10) | 0.30 |
| Hypertension | |  |  | 0.60 |
| 0 | 77 (46.39%) | 63 (47.37%) | 14 (42.42%) |  |
| 1 | 89 (53.61%) | 70 (52.63%) | 19 (57.58%) |  |
| Diabetes | |  |  | 0.90 |
| 0 | 109 (65.66%) | 87 (65.41%) | 22 (66.67%) |  |
| 1 | 57 (34.34%) | 46 (34.59%) | 11 (33.33%) |  |
| Chronic kidney disease | |  |  | 0.40 |
| 0 | 139 (83.73%) | 110 (82.71%) | 29 (87.88%) |  |
| 1 | 27 (16.27%) | 23 (17.29%) | 4 (12.12%) |  |
| Cerebrovascular disease | | |  | 0.30 |
| 0 | 158 (95.18%) | 125 (93.98%) | 33 (100.00%) |  |
| 1 | 8 (4.82%) | 8 (6.02%) | 0 (0.00%) |  |
| Mechanical ventilation | | |  | 0.006 |
| 0 | 47 (28.31%) | 44 (33.08%) | 3 (9.09%) |  |
| 1 | 119 (71.69%) | 89 (66.92%) | 30 (90.91%) |  |
| CRRT |  |  |  | 0.2 |
| 0 | 134 (80.72%) | 110 (82.71%) | 24 (72.73%) |  |
| 1 | 32 (19.28%) | 23 (17.29%) | 9 (27.27%) |  |
| Vasoactive drug use | |  |  | 0.002 |
| 0 | 71 (42.77%) | 64 (48.12%) | 7 (21.21%) |  |
| 1 | 95 (57.23%) | 69 (51.88%) | 26 (78.79%) |  |

Supplementary Table S8. Predictive performance of HGI, HbA1c, glucose, and SOFA for 28-day mortality.

| Model | AUC | 95%CI |
| --- | --- | --- |
| HGI | 0.561 | 0.53–0.59 |
| HbA1c | 0.535 | 0.51–0.56 |
| Glucose | 0.527 | 0.50–0.56 |
| SOFA | 0.546 | 0.52–0.57 |
| SOFA+HGI | 0.574 | 0.54–0.60 |
| SOFA+HbA1c | 0.558 | 0.53–0.59 |
| SOFA+Glucose | 0.548 | 0.52–0.58 |
| Base | 0.676 | 0.65–0.70 |
| Base+HGI | 0.680 | 0.65–0.71 |
| Base+HbA1c | 0.675 | 0.65–0.70 |
| Base+Glucose | 0.685 | 0.66–0.71 |

HGI / HbA1c / Glucose: Univariate models including only the Hemoglobin Glycation Index (HGI), glycated hemoglobin (HbA1c), or glucose, respectively.

SOFA: Univariate model including only the Sequential Organ Failure Assessment (SOFA) score.

SOFA+HGI / SOFA+HbA1c / SOFA+Glucose: Combined models integrating the SOFA score with HGI, HbA1c, or glucose, respectively.

Base (Base Model): A fully adjusted multivariable model including baseline demographic characteristics, comorbidities, and disease severity. The covariates are: age, gender, race, body mass index (BMI), SOFA score, hypertension, diabetes, cerebrovascular disease, and mechanical ventilation.

Base+HGI / Base+HbA1c / Base+Glucose: Extended models integrating HGI, HbA1c, or glucose into the Base model, respectively, to evaluate the incremental predictive value of these glycemic markers after adjusting for potential confounders.

Supplementary Table S9. Reclassification and discrimination statistics for 28-day mortality

| Models Compared | IDI (95% CI) | *P* -value | NRI (95% CI) | *P* -value |
| --- | --- | --- | --- | --- |
| Base+HGI vs. Base Model | 0.0033 (0.0002 to 0.0100) | 0.024 | 0.0290 (-0.0255 to 0.0869) | 0.228 |
| Base+HGI vs. Base + HbA1c | 0.0032 (-0.0012 to 0.0082) | 0.172 | 0.0450 (-0.0472 to 0.1250) | 0.363 |
| Base+HGI vs. Base + Glucose | -0.0014 (-0.0077 to 0.0052) | 0.587 | -0.0178 (-0.0872 to 0.0554) | 0.707 |


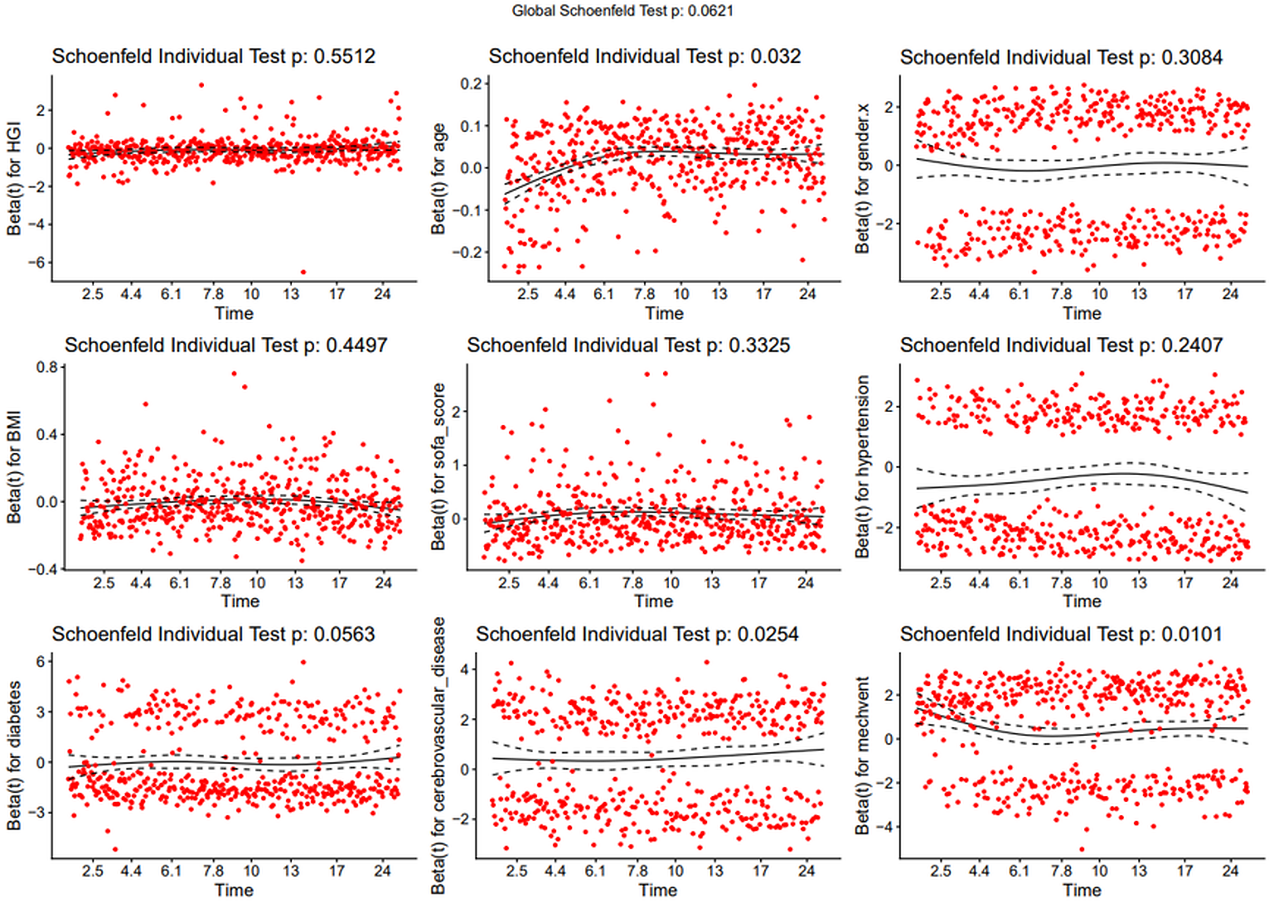
Figure S1. Schoenfeld residual plots for the Cox proportional hazards model with HGI as a continuous variable


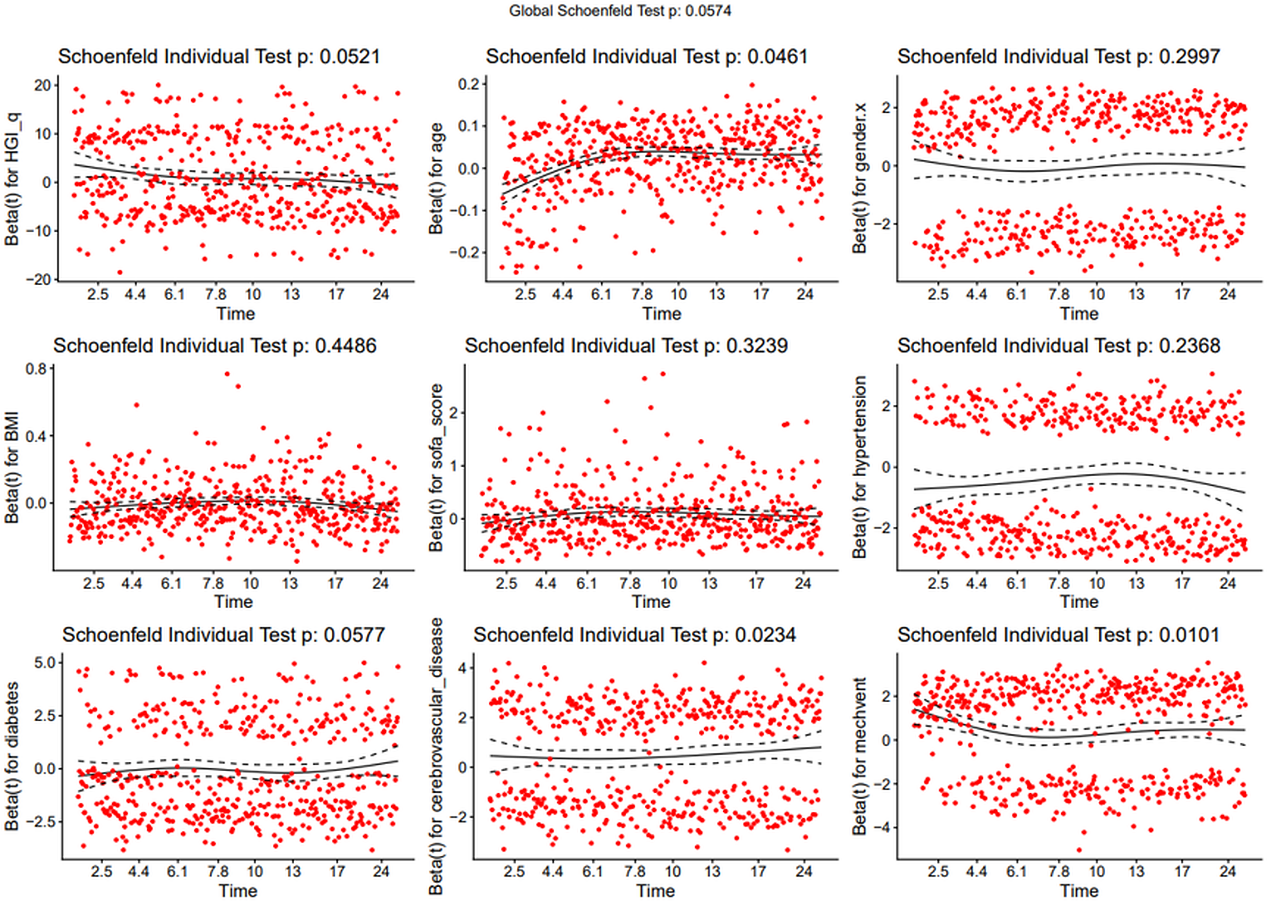


Figure S2. Schoenfeld residual plots for the Cox proportional hazards model with HGI quartiles


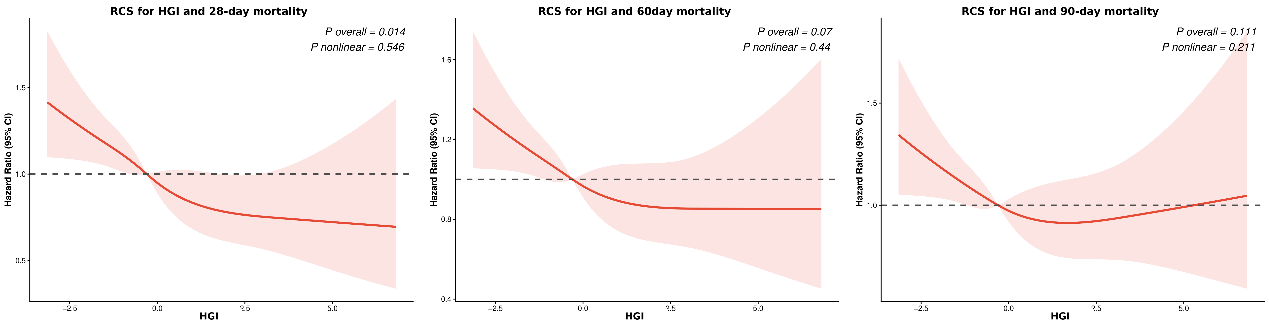


Supplementary Figure S3. Restricted cubic spline analysis of the association between continuous HGI and short-term mortality.
